# Evaluation of Clinical Characteristics and CT Decision Rules in Elderly Patients with Minor Head Injury: A Prospective Multicenter Cohort Study

**DOI:** 10.3390/jcm12030982

**Published:** 2023-01-27

**Authors:** Sophie M. Coffeng, Kelly A. Foks, Crispijn L. van den Brand, Korné Jellema, Diederik W. J. Dippel, Bram Jacobs, Joukje van der Naalt

**Affiliations:** 1 Department of Emergency Medicine, University of Groningen, University Medical Center Groningen, 9713 GZ Groningen, The Netherlands; 2 Department of Neurology, Erasmus MC University Medical Center Rotterdam, 3015 GD Rotterdam, The Netherlands; 3 Department of Emergency Medicine, Erasmus MC University Medical Center Rotterdam, 3015 GD Rotterdam, The Netherlands; 4 Department of Neurology, Haaglanden Medical Center, 2512 VA The Hague, The Netherlands; 5 Department of Neurology, University of Groningen, University Medical Center Groningen, 9713 GZ Groningen, The Netherlands

**Keywords:** minor head injury, intracranial lesions, CT decision rules, elderly patients, traumatic brain injury

## Abstract

Age is variably described as a minor or major risk factor for traumatic intracranial lesions after head injury. However, at present, no specific CT decision rule is available for elderly patients with minor head injury (MHI). The aims of this prospective multicenter cohort study were to assess the performance of existing CT decision rules for elderly MHI patients and to compare the clinical and CT characteristics of elderly patients with the younger MHI population. Thirty-day mortality between two age groups (cutoff ≥ 60 years), along with clinical and CT characteristics, was evaluated with four CT decision rules: the National Institute for Health and Care Excellence (NICE) guideline, the Canadian CT Head Rule (CCHR), the New Orleans Criteria (NOC), and the CT Head Injury Patients (CHIP) rule. Of the 5517 MHI patients included, 2310 were aged ≥ 60 years. Elderly patients experienced loss of consciousness (17% vs. 32%) and posttraumatic amnesia (23% vs. 31%) less often, but intracranial lesions (13% vs. 10%), neurological deterioration (1.8% vs. 0.2%), and 30-day mortality (2.0% vs. 0.1%) were more frequent than in younger patients (all *p* < 0.001). Elderly patients with age as their only risk factor showed intracranial lesions in 5% (NOC and CHIP) to 8% (CCHR and NICE) of cases. The sensitivity of decision rules in the elderly patients was 60% (CCHR) to 97% (NOC) when age was excluded as a risk factor. Current risk factors considered when evaluating elderly patients show lower sensitivity to identify intracranial abnormalities, despite more frequent intracranial lesions. Until age-specific CT decision rules are developed, it is advisable to scan every elderly patient with an MHI.

## 1. Introduction

Minor head injury (MHI) is a common and ever-growing cause of admission to emergency departments (EDs) worldwide [1]. MHI is the least severe category within the traumatic brain injury (TBI) spectrum and includes patients with and without clinical TBI characteristics such as loss of consciousness (LOC), posttraumatic amnesia (PTA), and focal neurological deficit [2]. The risk of sustaining a TBI increases with age, as elderly patients fall more frequently secondary to pre-existing frailty, dementia, and motor disorders [3,4,5]. Although these falls are most often from standing height, elderly individuals require more hospitalizations after TBIs and have higher mortality and morbidity rates [6]. It is important to gain more insight into the characteristics of this specific population of MHI patients with regard to the policy of evaluation, diagnosis, and treatment upon arrival to the ED. Elderly patients with MHI may have a different clinical presentation due to delayed clinical symptoms of intracranial hemorrhage, because of brain atrophy and specific intracranial abnormalities as a result of a higher vulnerability of vessels to rupture and lower-energy trauma mechanisms compared to younger patients [7,8,9]. Additionally, injury assessment at the ED may be more complicated in elderly patients due to coexistent mental impairment and additional pre-trauma intracranial pathology, which can influence the clinical presentation.

An important aspect of the evaluation of elderly patients with MHI at the ED is to determine the risk of intracranial injuries and, thus, the indication for a computed tomography (CT) scan of the head. Several decision rules for the application of head CT in patients with MHI at the ED have been developed with the purpose of detecting all relevant intracranial traumatic lesions while minimizing the number of unnecessary CT scans. The National Institute for Health and Care Excellence (NICE) guideline for head injury was developed for the full spectrum of MHI patients [10]. However, two other decision rules (the Canadian CT Head Rule (CCHR) and the New Orleans Criteria (NOC)) include only patients who have experienced loss of consciousness or posttraumatic amnesia [11,12]. The CT in Head Injury Patients (CHIP) rule was developed to also include patients without loss of consciousness or posttraumatic amnesia [13]. An issue with the aforementioned CT decision rules is that they are less representative of the current MHI population, since they were originally developed for a relatively young population (who most likely had significantly fewer comorbidities) and not specifically for the elderly. Moreover, the CT decision rules define the cutoff age—which is considered to be a risk factor—differently. The NOC and CHIP rules use a cutoff of 60 years as a major risk factor for intracranial pathology following a head injury, while the CCHR and the NICE guideline use 65 years as a major and minor risk factor, respectively [10,11,12,13]. The national guideline of the Netherlands is based on the CHIP rule, although age has been made a minor rather than a major criterion [14]. As a consequence, elderly patients with MHI only require a CT scan when they have at least one other risk factor—for example, LOC or the use of anticoagulant agents [10,14]. These varying CT scanning regimes warrant further investigation as to whether a CT scan of the head should be mandatory for every elderly MHI patient. This is especially relevant considering the higher medical costs, radiation exposure, and (over)crowding at the ED to which a more expansive CT scan regime could lead. Our research group previously completed an external validation of the CT decision rules in a cohort of Dutch patients after MHIs [15]. Although elderly patients were represented in this cohort, specific validation for this patient group was not performed.

Evidence-based guidelines on acute management for elderly patients with MHI are lacking. In most TBI studies, elderly patients are frequently excluded because of their past medical history [16,17]. Previous reviews highlight this gap in MHI knowledge and call for dedicated research into this growing population [9,18]. Only the NEXUS II criteria have been re-evaluated in a large population of elderly MHI patients [19]. Other recently published studies about decision rules were retrospective or included only a subgroup of elderly patients. For example, a new decision tree was investigated in elderly patients with ground-level falls, an amendment of the CCHR was examined for elderly patients in nursing homes with ground-level falls, and another CCHR study suggested increasing the age criterion to 75 years [20,21,22]. Therefore, the aim of this prospective study was to assess the performance of four commonly used CT decision rules for the elderly MHI population to determine whether it is necessary to use age as a major criterion for CT scanning. To this end, we evaluated the frequency of injury characteristics (clinical and traumatic), intracranial lesions, deterioration, and mortality of elderly patients with minor head injuries and compared these findings to their younger counterparts to discern more potential age-group-related differences in a defined cohort of patients with MHI.

## 2. Materials and Methods

### 2.1. Study Design and Setting

Data were derived from a prospective multicenter cohort study (the CREST study) conducted from March 2015 to December 2016 in nine EDs in the Netherlands [15]. The study cohort was divided into two groups—younger and elderly patients (defined by a cutoff of ≥60 years)—in accordance with both the CHIP and NOC decision rules [12,13]. We obtained institutional ethics and research board approval, and the need for informed consent was waived.

The inclusion criteria of the CREST study were as follows: blunt trauma to the head (all mechanisms), age ≥ 16 years, and presentation at the ED within 24 h after trauma with a Glasgow Coma Scale (GCS) score of 13–15. Patients were included irrespective of the presence of PTA or LOC. The exclusion criteria were as follows: GCS score of <13, age < 16 years, transfer from other hospitals, or any contraindication for obtaining a CT scan.

### 2.2. Data Collection

Consecutive patients with MHI were included by trained research physicians, who did not personally have contact with the patients. During the study period, the research physicians screened all of the patients who were seen at the emergency department daily and included the patient if eligible. This meant that any patient with blunt head trauma was included—not only the patients who had head trauma as their main complaint. After including the patient, the research physicians entered the required data into the digital study data management system, which included information about the injury, risk factors, and outcomes. The attending clinicians were trained to fill out forms containing the relevant risk factors or they were asked to enter information concerning the risk factors in the patient’s electronic medical record. Refer to Foks et al. for more details about the risk factors [15].

The participating centers used the Dutch national guideline based on the CHIP rule, with age defined as a minor risk factor [14]. The CT scans were interpreted by (neuro)radiologists who were aware of the patient’s medical history and clinical findings.

The clinical risk factors were derived from the criteria used in the NICE, CCHR, NOC, and/or CHIP decision rules [10,11,12,13]. The NOC and CCHR rules were developed for specific MHI populations, with the NOC excluding MHI patients with GCS scores of <15, while CCHR excluded patients who used coumarin medications, had a history of bleeding disorders, or had an obvious skull fracture. In order to investigate the entire MHI population, these original exclusion criteria were added as additional major risk factors.

One of the risk factors is ‘dangerous mechanism’, which includes the following (high-energy) trauma mechanisms: pedestrian or cyclist versus vehicle, ejected from vehicle, fall from elevation (>1 m or five stairs), or an equivalent mechanism.

Information on hospital admission, clinical neurological deterioration, neurosurgical intervention, mortality, moment of discharge, and 30-day follow-up on neurosurgical interventions was retrieved from the electronic medical records.

Large intracranial lesions on the head CT were defined as epidural hematoma, subdural hematoma (mass), contusion(s) (mass), depressed skull fracture, and any lesion resulting in a midline shift or compression of the basal cisterns [18]. The remaining traumatic lesions were defined as small intracranial lesions. ‘Any intracranial lesion’ included all small and large traumatic intracranial lesions, while ‘Relevant intracranial lesion’ included large lesions and small lesions that needed neurosurgical intervention within 30 days. Moreover, patients who deteriorated or died during admission because of their intracranial lesion were labelled as having a relevant intracranial lesion.

We completed a subgroup analysis with the aim of evaluating whether elderly patients who did not meet a scanning criterion except for their age could have intracranial lesions. We examined two subpopulations of elderly patients: a group of patients with age as their only risk factor for a CT scan, and a group of patients with a GCS score of 15 with or without one or more clinical TBI risk factors (i.e., posttraumatic amnesia, loss of consciousness, retrograde amnesia, or focal neurological deficit) after MHI.

### 2.3. Outcomes

The primary outcome measure was the performance of the current CT decision rules for the elderly MHI population, depicted by the frequency and type of lesion defined as ‘Any intracranial lesion’ or a ‘Relevant intracranial lesion’ on the CT scan. The secondary outcome measures were deterioration during admission, neurosurgical intervention, and 30-day mortality.

### 2.4. Statistical Analysis

SPSS Data Editor 23.0 (IBM SPSS Statistics, SPSS Inc., Chicago, IL, USA) was used for statistical analysis. Demographic and trauma characteristics were displayed as means and standard deviations or medians and interquartile ranges for continuous variables, and as frequencies and percentages for categorical variables. Differences between younger and elderly patients were tested with Student’s *t*-test or the nonparametric Mann–Whitney U-test. Nominal statistics were performed with Pearson’s *X*^2^ test. A two-tailed probability < 0.05 was considered to be significant.

To minimize potential bias, missing data were assumed to be missing at random. Therefore, missing data were imputed on the basis of all available clinical risk factors with R version 3.3.2 (R Foundation for Statistical Computing), using multiple imputation (*n* = 5) with the ‘multivariable imputation by chained equations’. Additionally, outcomes (i.e., head CT abnormalities) could not be observed in patients without a CT scan. Therefore, for these patients, the expected outcomes (‘Any intracranial lesion’ and ‘Relevant intracranial lesion’) were imputed on the basis of their risk factors with multiple imputation to avoid selection bias and, thus, yield unbiased estimates of sensitivity and specificity [23]. Specifically, without imputation, this might result in an overestimation of sensitivity and underestimation of specificity for all of the rules.

To assess the performance of the CT decision rules in combination with investigating the value of the risk factor (age ≥ 60 years), we excluded this age criterion from the four most commonly used CT decision rules and calculated the sensitivity, specificity, positive predictive value, and negative predictive value of the adapted decision rules in elderly patients. For the subgroup analysis of the predictive value of clinical TBI characteristics (i.e., maximum GCS score in combination with PTA, LOC, retrograde amnesia, or focal neurological deficit) in elderly patients with a maximal GCS score, the sensitivity, specificity, and positive and negative predictive values were also calculated. Sensitivity was calculated by dividing the number of patients with clinical TBI characteristic(s) and intracranial lesions (any or relevant) by the total number of patients with intracranial lesions (any or relevant). Specificity was calculated by dividing the number of patients without intracranial lesions and no clinical TBI characteristic(s) by the total number of patients without intracranial lesions. The positive predictive value was calculated by dividing the number of patients with intracranial lesions and clinical TBI characteristic(s) by the total number of patients with clinical TBI characteristic(s). The negative predictive value was calculated by dividing the number of patients without intracranial lesions and with no clinical TBI characteristic(s) by the total number of patients without clinical TBI characteristic(s).

## 3. Results

**Figure 1:**
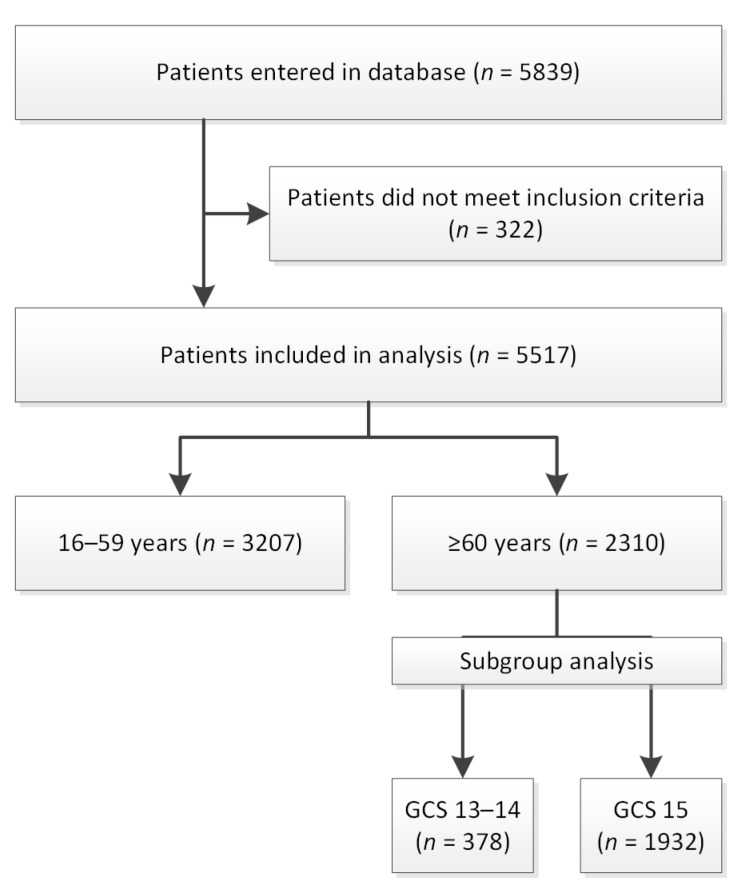
Study flowchart. GCS = Glasgow Coma Scale.

In total, 5517 patients were included for further analysis; 3207 patients were aged < 60 years, and 2310 patients were aged ≥ 60 years (Figure 1).

### 3.1. Demographic and Clinical Characteristics

Elderly patients experienced loss of consciousness (17% vs. 32%, *p* < 0.001), posttraumatic amnesia (23% vs. 31%, *p* < 0.001), and posttraumatic headache (25% vs. 36%, *p* < 0.001) less often than the younger patients. The most common trauma mechanism in the elderly population was a fall from standing height (60% vs. 19%, *p* < 0.001), which mostly occurred at home (50% vs. 18%, *p* < 0.001) (Table 1). Elderly patients were admitted to the hospital more frequently than younger patients (44% vs. 30%; *p* < 0.001), with neurosurgical intervention taking place in 11 (0.5%) elderly patients and in 8 (0.2%) younger patients (*p* = 0.156).

### 3.2. Computed Tomography after MHI in Elderly Patients

A head CT was performed in 96% (*n* = 2210) of the elderly and 78% of the younger patient group (*n* = 2492), as not all patients met the local scanning criteria. Both ‘Any intracranial lesion’ (13% (*n* = 284) vs. 10% (*n* = 243); *p* < 0.001) and ‘Relevant intracranial lesions’ (3% (*n* = 77) vs. 2% (*n* = 51); *p* < 0.001) were more frequently seen in elderly patients. Elderly patients with any intracranial lesion had acute subdural hematoma (SDH) (45% (*n* = 129) vs. 29% (*n* = 71); *p* = 0.007) more often and epidural hematoma (6% (*n* = 16) vs. 15% (*n* = 36); *p* = 0.002) less often compared to the younger group. The occurrence of other traumatic CT characteristics, such as subarachnoid hemorrhage and contusions, was comparable between both age groups. The percentage of ‘Any intracranial lesion’ in patients with anticoagulant medication was almost twice as high in elderly patients compared to their younger counterparts, although this was not statistically significant (8% (*n* = 48) vs. 5% (*n* = 2), *p* = 0.435). The same applied to antiplatelet therapy (14% (*n* = 90) vs. 11% (*n* = 8), *p* = 0.450) (Table 2 and Table S1).

### 3.3. CT Decision Rules in Elderly Patients

After excluding age as a risk factor, the sensitivity for identifying an elderly patient with any intracranial lesion according to the four decision rules ranged from 60.1% (CCHR) to 97.2% (NOC). For identifying a relevant intracranial lesion, the sensitivity was the lowest using the CCHR (76.6%) and 100% using the NOC (Table 3).

We also examined a subgroup of elderly patients without risk factors for intracranial lesions according to the commonly used CT decision rules, except for being aged 60 years or older. Of these patients with age as their only risk factor, 5–8% had any intracranial lesion and 0.5–1% had a relevant intracranial lesion, depending on which CT decision rule was used (Table 4).

In the group of patients with a GCS score of 15 and with any intracranial lesion, 59 of 177 (33%) patients had no other TBI characteristics (e.g., posttraumatic amnesia, loss of consciousness, retrograde amnesia, or focal neurological deficit) (Table 5). Almost half of all elderly patients with a relevant intracranial lesion (*n* = 32) had a maximal GCS score at the ED, while seven patients had none of these clinical TBI characteristics.

### 3.4. Neurological Deterioration and Deceased Elderly Patients

During admission, 53 (2.3%) elderly patients deteriorated, of whom 42 (1.8%) primarily deteriorated because of their MHI. This deterioration rate was nine times higher compared to the younger cohort (eight patients (0.2%); *p* < 0.001). All of these patients with neurological deterioration initially had intracranial lesions, with a small lesion in 20 patients, constituting 8.8% of all elderly patients with a small lesion. A large lesion was present in 22 patients, constituting 39% of all elderly patients with a large lesion. The most common intracranial lesions in deteriorating patients were acute SDH (48%) and traumatic subarachnoid hemorrhage (43%). The remaining 11 patients, who deteriorated due to other causes (such as systemic traumatic abnormalities or non-traumatic diseases such as heart failure), had an initial normal head CT scan without new traumatic intracranial lesions on repeated CT scans.

A total of 46 (2%) elderly patients died at the ED or during hospital admission, compared to 2 (0.1%) of the younger patients (*p* < 0.001). Of the deceased patients, 12 elderly patients died directly as result of their MHI, with an initial large lesion in 11 patients. Acute SDH was the most common intracranial lesion (10 patients). Eight deceased patients (67%) used anticoagulants and/or antiplatelet therapy. In seven patients (58%), the trauma mechanism was a fall from standing height.

## 4. Discussion

This study shows that elderly patients aged 60 years or older with MHI more frequently have traumatic CT lesions as well as a higher frequency of neurological deterioration and a higher mortality rate when compared to younger patients. Despite this, the elderly more frequently presented without clinical signs of MHI. As a consequence, the commonly used risk factors denoted in several CT decision rules for MHI clearly show a lower sensitivity to identify traumatic intracranial abnormalities in elderly patients. Therefore, age should be regarded as a major criterion for performing a head CT.

In elderly patients with MHI, traumatic intracranial lesions on CT are common when compared to their younger counterparts. In our cohort, 13% of patients aged 60 years or older had any intracranial lesion and 3% had a relevant intracranial lesion. Although previous studies reported divergent percentages in elderly patients (2% to 21%), our study is one of the first prospective studies with a substantial sample size [11,12,24,25,26,27]. In addition to having more frequent intracranial lesions after MHI, elderly patients also present with different types of traumatic lesions compared to younger patients. In particular, they are more at risk of developing subdural hematomas, most likely due to age-related changes in vessels, resulting in higher vulnerability to rupture [28,29]. Acute subdural hematomas result in worse outcomes, most likely related to underlying cortical (ischemic) brain damage [30,31]. In addition to the presence of potentially more serious traumatic intracranial lesions, elderly patients with MHI also have an increased risk of clinical neurological deterioration and a higher 30-day mortality rate. In our elderly patient group, the neurological deterioration rate was 9 times higher and the mortality rate was as much as 20 times higher when compared to their younger counterparts.

Regarding the role of the mechanism of trauma, we found that elderly patients with MHI sustained low-energy trauma more frequently. Falls from standing position were the most common trauma mechanism and were reported in as many as 34% of patients with a relevant intracranial lesion. This finding is consistent with those of previous studies [32,33]. Our results show that although dangerous mechanisms (e.g., falls from height or traffic accidents) are well-known risk factors for TBI, they did not seem to be associated with relevant intracranial lesions in our cohort of elderly patients with MHI. This finding might indicate that the type of trauma mechanism—especially the distinction between low- and high-energy trauma—is not sufficient to estimate the severity of the impact and the risk of intracranial lesions in elderly MHI patients. As a consequence, clinicians must be aware that low-energy accidents can also lead to significant intracranial lesions in this specific patient category.

As elderly patients develop intracranial injury more frequently after MHI than younger patients, it is essential to determine which factors can identify patients at risk of intracranial traumatic abnormalities. However, the assessment of elderly patients with MHI at the ED is challenging, as clinical characteristics in these patients are often subtle, absent, or difficult to assess because of pre-existing physical and cognitive problems [18]. In a subgroup of our cohort without the well-known accompanying risk factors for intracranial traumatic injury, head CT still showed traumatic intracranial lesions in 5–8% of patients. Moreover, one-third of the elderly patients with an intracranial lesion and a maximal GCS score at the ED did not have clinical signs of TBI. Our findings confirm those of Rathlev et al., who investigated the NEXUS II criteria in elderly patients—one of the CT decision rules that we did not include in our analysis [19]. They found that 2.2% of the elderly patients with an MHI had no evidence of clinically significant skull fracture, neurological deficit, or altered levels of consciousness. In that study, three patients with significant injury did not manifest any of the 16 clinical findings commonly used to assess MHI in patients. GCS scores, along with the other well-known risk factors, might therefore inadequately reflect the severity of MHI in elderly patients. Consequently, despite limited clinical signs of intracranial injury—i.e., a concealed clinical presentation—elderly patients with MHI can certainly have traumatic intracranial lesions.

As GCS score, trauma mechanism, and other well-known risk factors are not sufficient to predict intracranial lesions in elderly patients, a relevant question for the application of CT decision rules is whether it is necessary to perform a head CT scan in every elderly patient with MHI presenting at the ED. The CCHR, NOC, the original CHIP rule, and the NEXUS II criteria already advise scanning every elderly patient, as age is a major criterion [10,11,12,13,34]. This is in contrast to the national Dutch, NICE, and Scandinavian guidelines [14,35]. The latter guideline does not include age as a risk factor, stating that its predictive ability is only moderate. However, not every small intracranial lesion has clinical consequences; therefore, it is particularly important to detect relevant intracranial lesions [11,12,13]. In clinical practice, it has been suggested to use the same scanning criteria for elderly patients as for the younger population, and to exclude the risk factor of age 60 years or older [26]. If we had applied this recommendation to our study cohort, 5–8% of the elderly patients with any intracranial lesion and 1% of those with a relevant intracranial lesion, including one patient who needed neurosurgical intervention, would not have been scanned on initial presentation. Ideally, a sensitivity of 100% is desired in order to detect every relevant intracranial lesion. If we want to achieve this 100% sensitivity, based on our study population, every patient aged 60 years or older who presents at the ED after a head injury should undergo a CT scan. Consequently, this will increase medical costs, radiation load, and crowding at the ED, and the question is whether that can be justified. A previous study on the cost-effectiveness of CT scanning in patients with MHI has shown that the sensitivity of a prediction rule should be at least 97% for identifying potential neurosurgical lesions in order for it to be cost-effective [36]. This threshold may be too low for elderly patients, as the development of malignancy as a consequence of ionizing radiation is negligible among elderly patients with reduced lifespans. It may thus be more appropriate to increase the sensitivity to 99%. Our analyses of the different CT decision rules in elderly patients have shown that none of the CT decision rules meets this requirement if we exclude age as a criterion.

Some limitations of this study should be mentioned. Firstly, in this study, only the risk factors relevant to the four most commonly used CT decision rules were collected. Other risk factors that are specific to the elderly age group might have been missed, such as, pre-injury frailty and pre-injury health status. Secondly, the follow-up was limited to 30 days for neurosurgical intervention and mortality. Patients who were discharged with a normal head CT scan could theoretically have developed an intracranial abnormality over time. Nevertheless, this is very rare at such a late stage [37]. Thirdly, only patients with MHI who presented to the ED were included in this study, and non-hospitalized patients such as those who were evaluated at the general practitioner’s office were not included. Finally, not all patients with MHI in our study underwent a head CT scan—especially young patients who did not fulfil the national scanning criteria. To overcome this problem, missing data were imputed to avoid possible false negative findings. Although imputation might be considered suboptimal, when not applied—and if we assigned all patients without a CT scan as non-low risk—the sensitivity of the four decision rules would be overestimated and the specificity would be underestimated. In addition, the CHIP rule was mainly used in the study centers to decide whether a CT scan was necessary, and without imputation we would introduce a larger bias in favor of the CHIP decision rule, because of possible missed lesions that would have been detected by other decision rules. With the imputation of outcomes, 25 additional patients had any intracranial lesion, and there was one extra relevant intracranial lesion in the <60 years group. Imputation will therefore not have had much influence on our results. Despite these limitations, our findings are based on a prospective multicenter study with a considerable number of patients. Therefore, we deem it likely that our results are representative of the overall population of elderly patients with MHI.

In summary, this large prospective study provides the opportunity to assess the performance of four commonly used CT decision rules in elderly patients with MHI. We found that intracranial lesions were more often present in elderly patients with a concealed clinical presentation. The results of our study, combined with the earlier findings of the NEXUS II study in which elderly patients with MHI were evaluated, provide substantial scientific evidence that a concealed clinical presentation is common among older patients despite significant traumatic intracranial injury that may only be detected by CT imaging. Thereby, our paper underlines the need to preserve age as an independent risk factor for significant intracranial lesions, as suggested in the NOC, CCHR, original CHIP rule, and NEXUS II guidelines, until more specific clinical and injury-related characteristics or biomarkers become available to detect traumatic intracranial injury in elderly patients. This also implies that for local and national guidelines, where age is currently a minor criterion, an age of 60 years and older should be a major scanning criterion in patients with MHI.

## Figures and Tables

**Table 1:** Demographics and trauma characteristics of younger versus older patients at the ED after MHI.

	No. (%)			
	<60 Years *n* = 3207	≥60 Years *n* = 2310	*p*-Value	Missing Values No. (%)
Sex (female)	1079 (34)	1218 (53)	<0.001	0 (0.0)
Age (years) (mean (SD))	36 (13)	76 (10)	<0.001	0 (0.0)
Initial GCS score < 15	536 (17)	378 (16)	0.730	0 (0.0)
GCS deterioration				
- 1 point	17 (0.5)	24 (1.0)	0.092	31 (0.5)
- ≥2 points	9 (0.3)	9 (0.4)	0.770	
Posttraumatic amnesia	980 (31)	529 (23)	<0.001	632 (11)
≥4 h	145 (5)	63 (3)	<0.001	
Loss of consciousness	1016 (32)	382 (17)	<0.001	851 (15)
Amnesia before impact (≥30 min)	126 (4)	75 (3)	0.018	932 (17)
Posttraumatic headache	1163 (36)	581 (25)	<0.001	811 (15)
Vomiting	266 (8)	142 (6)	0.026	81 (1)
- ≥2 episodes	128 (4)	70 (3)	0.026	
Alcohol/drug intoxication	1024 (32)	277 (12)	<0.001	99 (2)
Anticoagulation treatment	40 (1)	578 (25)	<0.001	35 (0.6)
Bleeding or clotting disorder	24 (0.7)	29 (1)	0.137	37 (0.7)
Visible trauma above the clavicle	2381 (74)	1899 (83)	<0.001	40 (0.7)
Contusion of the skull (excl. face)	1497 (47)	1434 (62)	<0.001	33 (0.6)
Suspected open or depressed skull fracture	18 (0.6)	7 (0.3)	0.355	45 (0.8)
Any sign of basal skull fracture	97 (3)	94 (4)	0.071	37 (0.7)
Focal neurological deficit	68 (2)	67 (3)	<0.001	150 (3)
Posttraumatic seizure	32 (1)	13 (0.6)	0.168	86 (2)
Dangerous trauma mechanism				62 (1)
- Pedestrian struck by vehicle	55 (2)	24 (1)	<0.001	
- Cyclist struck by vehicle	147 (5)	63 (3)	<0.00	
- Occupant ejected	156 (5)	74 (3)	<0.001	
- Fall from elevation *	390 (12)	350 (15)	0.001	
Fall from standing position	612 (19)	1383 (60)	<0.001	21 (0.4)
Traumatic CT findings				815 (15) **
- Any intracranial lesion	243 (10)	284 (13)	<0.001	
- Relevant intracranial lesion	51 (2)	77 (3)	<0.001	
30-Day mortality	2 (0.1)	46 (2)	<0.001	

Abbreviations: ED = emergency department, GCS = Glasgow Coma Scale, IQR = interquartile range, ISS = Injury Severity Score, MHI = minor head injury, SD = standard deviation. Values are listed as number (%) unless otherwise specified. * Fall from > 3 feet/5 stairs. ** CT was not performed, as these patients did not meet the local scanning criteria.

**Table 2:** Demographics and trauma characteristics of intracranial lesions after MHI in elderly patients (*n* = 2210).

		No. (%)	
	Any Intracranial Lesion *n* = 284	Relevant Intracranial Lesion ^a^ *n* = 77	Normal CT Scan *n* = 1926
Sex (female)	128 (44) *	24 (31) **	1031 (53)
Age (years) mean (SD)	75 (10) *	77 (10)	77 (10)
Initial GCS score < 15	109 (38) *	45 (58) **	266 (14)
GCS deterioration			
- 1 point	4 (1)	1 (1)	20 (1)
- ≥2 points	6 (2)	4 (5)	3 (0.2)
Posttraumatic amnesia	179 (63) *	62 (81) **	501 (26)
- ≥4 h	42 (15) *	22 (29) **	47 (2)
Loss of consciousness	162 (57) *	50 (65) **	583 (30)
- >15 min	13 (4) *	2 (3)	20 (1)
Posttraumatic headache	111 (39) *	32 (42) **	577 (30)
Amnesia before impact (≥ 30 min)	48 (17) *	22 (29) **	65 (3)
Vomiting	39 (14) *	18 (23) **	106 (6)
- ≥ 2 episodes	22 (8) *	11 (14) **	48 (3)
Alcohol/drug intoxication	31 (11)	6 (8)	251 (13)
Anticoagulation treatment	48 (17) *	18 (23)	529 (27)
Antiplatelet therapy	90 (32)	29 (39)	536 (28)
Bleeding or clotting disorder	3 (1)	1 (1)	26 (1)
Visible trauma to the head (excl. face)	188 (66)	50 (65)	1211 (63)
Visible trauma to the face	87 (31)	24 (31)	562 (29)
Suspected open or depressed skull fracture	4 (1)	1 (1)	3 (0.2)
Any sign of basal skull fracture	35 (12) *	12 (16) **	61 (3)
Focal neurological deficit	20 (7) *	9 (12) **	47 (2)
Posttraumatic seizure	4 (1)	2 (3)	12 (0.6)
Dangerous trauma mechanism			
- Pedestrian struck by vehicle	5 (2)	1 (1)	18 (0.9)
- Cyclist struck by vehicle	17 (6) *	6 (8) **	46 (2)
- Occupant ejected	16 (6) *	4 (5)	60 (3)
- Fall from height	67 (24) *	22 (29) **	279 (15)
Fall from standing position	113 (40) *	26 (34) **	1223 (64)

Abbreviations: CT = computed tomography, GCS = Glasgow Coma Scale, MHI = minor head injury, SD = standard deviation. Values are listed as number (%) unless otherwise specified; * *p* < 0.05, significant difference between any traumatic abnormality and normal CT scan; ** *p* < 0.05, significant difference between relevant traumatic lesion and normal CT scan. ^a^ Relevant intracranial lesions are defined as (potential) neurosurgical CT abnormalities, deterioration, or death during admission. Missing values were corrected by multiple imputation.

**Table 3:** Performance of the four commonly used decision rules in 2310 elderly patients with MHI when age was excluded as a risk factor.

CT Decision Rule	Positive Outcome (*n*)	Negative Outcome (*n*)	Sensitivity (% (95% CI))	Specificity (% (95% CI))	Positive Predictive Value (% (95% CI))	Negative Predictive Value (% (95% CI))
**NOC**						
Any intracranial lesion						
- NOC positive	278	1882	97.2	7	12.9	94.7
- NOC negative	8	142	(95.3 to 99.1)	(5.9 to 8.1)	(11.5 to 14.3)	(91.1 to 98.3)
Relevant intracranial lesion						
- NOC positive	77	2083	100	6.7	3.6	100
- NOC negative	0	150	(100 to 100)	(5.7 to 7.7)	(2.8 to 4.4)	(100 to 100)
**CCHR**						
Any intracranial lesion						
- CCHR positive	172	676	60.1	66.6	20.3	92.2
- CCHR negative	114	1348	(54.4 to 65.8)	(64.5 to 68.7)	(17.6 to 23.0)	(90.8 to 93.6)
Relevant intracranial lesion						
- CCHR positive	59	789	76.6	64.7	7	99
- CCHR negative	18	1444	(67.1 to 86.1)	(62.7 to 66.7)	(5.3 to 8.7)	(98.5 to 99.5)
**CHIP rule**						
Any intracranial lesion						
- CHIP positive	249	1318	87.1	34.9	15.9	95
- CHIP negative	37	706	(83.2 to 91.0)	(32.8 to 40.0)	(14.1 to 17.7)	(93.4 to 96.6)
Relevant intracranial lesion						
- CHIP positive	73	1494	94.8	33.1	4.7	99.5
- CHIP negative	4	739	(89.8 to 99.8)	(31.1 to 35.1)	(3.7 to 5.7)	(99.0 to 100)
**NICE**						
Any intracranial lesion						
- NICE positive	194	952	67.8	53	16.9	92.1
- NICE negative	92	1072	(62.4 to 73.2)	(50.8 to 55.2)	(14.7 to 19.1)	(90.6 to 93.6)
Relevant intracranial lesion						
- NICE positive	65	1081	84.4	51.6	5.7	92.9
- NICE negative	12	1152	(76.3 to 92.5)	(49.5 to 53.7)	(4.4 to 7.1)	(91.4 to 94.4)

Abbreviations: CCHR = Canadian CT Head Rule, CHIP = CT in Head Injury Patients, CI = confidence interval, MHI = minor head injury, NOC = New Orleans Criteria. NICE = National Institute for Health and Care Excellence guideline for head injury. Missing values were corrected by multiple imputation.

**Table 4:** Subgroup analysis of elderly patients with age as their only risk factor after MHI.

	Elderly with Age as Their Only Risk Factor (*n* (%))	Number Needed to Scan
**NOC**	*n* = 150 **	
Any intracranial lesion	7 (5)	21
Relevant intracranial lesion *	1 (0.6)	150
Neurosurgical intervention	0 (0)	-
**CCHR**	*n* = 1462 **	
Any intracranial lesion	112 (8)	13
Relevant intracranial lesion *	19 (1)	77
Neurosurgical intervention	1 (0.1)	1462
**CHIP rule**	*n* = 743 **	
Any intracranial lesion	36 (5)	21
Relevant intracranial lesion *	4 (0.5)	186
Neurosurgical intervention	0 (0.0)	-
**NICE**	*n* = 1164 **	
Any intracranial lesion	92 (8)	12
Relevant intracranial lesion *	12 (1)	97
Neurosurgical intervention	1 (0.1)	1164

Abbreviations: CCHR = Canadian CT Head Rule, CHIP = CT in Head Injury Patients, MHI = minor head injury, NOC = New Orleans Criteria. NICE = National Institute for Health and Care Excellence guideline for head injury. * Relevant intracranial lesions are defined as (potential) neurosurgical CT abnormalities, deterioration, or death during admission. ** Numbers of patients were not similar per decision rule, because each decision rule has slightly different defined risk factors.

**Table 5:** Subgroup analysis of the predictive value of clinical TBI characteristics * in elderly patients with a maximal GCS score ** (*n* = 1932).

	Any Intracranial Lesion (*n* = 177) % (95% CI)	Relevant Intracranial Lesion ^†^ (*n* = 32) % (95% CI)
Sensitivity	67 (60–74)	78 (64–92)
Specificity	65 (63–67)	63 (62–65)
Positive predictive value	16 (13–19)	3 (2–5)
Negative predictive value	95 (94–96)	99 (99–100)

Abbreviations: CI = confidence interval. * Clinical TBI (traumatic brain injury) characteristics were defined as any one of the following: posttraumatic amnesia, loss of consciousness, retrograde amnesia, or focal neurologic deficit. ** Maximal GCS score = Glasgow Coma Scale score of 15. ^†^ Relevant intracranial lesions are defined as (potential) neurosurgical CT abnormalities, deterioration, or death during admission.

## Data Availability

Anonymized patient-level data are available upon reasonable request after contact with K. Foks: k.foks@erasmusmc.nl.

## Supplementary Materials

The following supporting information can be downloaded at: https://www.mdpi.com/article/10.3390/jcm12030982/s1, Table S1: Demographics and trauma characteristics of intracranial lesions after MHI in patients aged 16–59 years.
