# Supplementary material for: Evaluation of Clinical Characteristics and CT Decision Rules in Elderly Patients with Minor Head Injury: A Prospective Multicenter Cohort Study
Source: J Clin Med. 2023 Jan 27;12(3):982. doi: 10.3390/jcm12030982 (PMC9917997; doi:10.3390/jcm12030982)
Supplement: Supplementary file 1 [file jcm-12-00982-s001.zip › jcm-2089511-supplementary.pdf]

Supplemental Table S1: Demographics and trauma characteristics of intracranial lesions after MHI in patients aged 16-59 years.

	No. (%)		
	**Any intracranial lesion n= 243**	**Relevant intracranial lesion[a] n= 51**	**Normal CT scan n= 2249**
**Sex (Female)**	65 (27)*	10 (20)**	771 (34)
**Age (years) mean (SD)**	39 (13)	38 (12)	37 (13)
**Initial GCS score < 15**	100 (41)*	28 (55)**	429 (19)
**GCS deterioration**			
**- 1 point**	3 (1.2)	1 (2)	26 (0.7)
**- ≥ 2 points**	3 (1.2)*	2 (4)**	6 (0.3)
**Posttraumatic amnesia**	175 (72)*	42 (82)**	946 (42)
**- ≥ 4 hours**	41 (17)*	11 (22)**	136 (6)
**Loss of consciousness**	140 (70)*	38 (75)**	1068 (48)
**- > 15 min**	15 (6)*	5 (10)**	19 (0.8)
**Posttraumatic headache**	117 (48)	23 (45)	1025 (46)
**Amnesia before impact (≥ 30 minutes)**	49 (20)*	18 (35)**	153 (7)
**Vomiting**	26 (11)	4 (8)	236 (11)
**- ≥ 2 episodes**	14 (6)	3 (6)	115 (5)
**Alcohol/drugs intoxication**	88 (36)	14 (28)	851 (38)
**Anticoagulation treatment**	2 (0.8)	1 (2)	39 (2)
**Antiplatelet therapy**	8 (3)	0 (0)	64 (3)
**Bleeding or clotting disorder**	0 (0)	0 (0)	23 (1)
**Visible trauma to the head (excl. face)**	151 (62)*	37 (72)**	1041 (46)
**Visible trauma to the face**	91 (37)	18 (35)	862 (38)
**Suspected open or depressed skull fracture**	13 (5)*	3 (6)**	5 (0.2)
**Any sign of basal skull fracture**	43 (18)*	12 (24)**	50 (2)
**Focal neurological deficit**	9 (4)	3 (6)	57 (3)
**Posttraumatic seizure**	7 (3)*	2 (4)	22 (1)
**Dangerous trauma mechanism**			
- pedestrian struck by vehicle	7 (3)	2 (4)	43 (2)
- cyclist struck by vehicle	19 (8)	6 (12)	122 (5)
- occupant ejected	20 (8)	4 (8)	133 (6)
- fall from height	60 (25)*	16 (31)**	299 (13)
**Fall from standing position**	36 (15)	7 (14)	460 (21)

*Abbreviations: CT = computed tomography, GCS = Glasgow Coma Scale, MHI = minor head injury, SD = Standard deviation. Values are listed as number (%) unless otherwise specified. * p< 0.05 significant difference between any traumatic abnormality and normal CT scan. ** p< 0.05 significant difference between relevant traumatic abnormality and normal CT scan. .[a]Relevant intracranial lesions is defined as (potential) neurosurgical CT abnormalities, deterioration or deceased during admission. Missing values corrected by multiple imputation.*
